# Immunoprecipitation of RNA–DNA hybrid interacting proteins in *Trypanosoma brucei* reveals conserved and novel activities, including in the control of surface antigen expression needed for immune evasion by antigenic variation

**DOI:** 10.1093/nar/gkad836

**Published:** 2023-10-16

**Authors:** Mark J Girasol, Emma M Briggs, Catarina A Marques, José M Batista, Dario Beraldi, Richard Burchmore, Leandro Lemgruber, Richard McCulloch

**Affiliations:** University of Glasgow, College of Medical, Veterinary and Life Sciences, School of Infection and Immunity, Wellcome Centre for Integrative Parasitology, Glasgow, UK; University of the Philippines Manila, College of Medicine, Manila, Philippines; University of Glasgow, College of Medical, Veterinary and Life Sciences, School of Infection and Immunity, Wellcome Centre for Integrative Parasitology, Glasgow, UK; University of Edinburgh, Institute for Immunology and Infection Research, School of Biological Sciences, Edinburgh, UK; University of Glasgow, College of Medical, Veterinary and Life Sciences, School of Infection and Immunity, Wellcome Centre for Integrative Parasitology, Glasgow, UK; University of Glasgow, College of Medical, Veterinary and Life Sciences, School of Infection and Immunity, Wellcome Centre for Integrative Parasitology, Glasgow, UK; University of Glasgow, College of Medical, Veterinary and Life Sciences, School of Infection and Immunity, Wellcome Centre for Integrative Parasitology, Glasgow, UK; University of Glasgow, College of Medical, Veterinary and Life Sciences, School of Infection and Immunity, Wellcome Centre for Integrative Parasitology, Glasgow, UK; University of Glasgow, College of Medical, Veterinary and Life Sciences, School of Infection and Immunity, Wellcome Centre for Integrative Parasitology, Glasgow, UK; University of Glasgow, College of Medical, Veterinary and Life Sciences, School of Infection and Immunity, Wellcome Centre for Integrative Parasitology, Glasgow, UK

## Abstract

RNA–DNA hybrids are epigenetic features of genomes that provide a diverse and growing range of activities. Understanding of these functions has been informed by characterising the proteins that interact with the hybrids, but all such analyses have so far focused on mammals, meaning it is unclear if a similar spectrum of RNA–DNA hybrid interactors is found in other eukaryotes. The African trypanosome is a single-cell eukaryotic parasite of the Discoba grouping and displays substantial divergence in several aspects of core biology from its mammalian host. Here, we show that DNA–RNA hybrid immunoprecipitation coupled with mass spectrometry recovers 602 putative interactors in *T. brucei* mammal- and insect-infective cells, some providing activities also found in mammals and some lineage-specific. We demonstrate that loss of three factors, two putative helicases and a RAD51 paralogue, alters *T. brucei* nuclear RNA–DNA hybrid and DNA damage levels. Moreover, loss of each factor affects the operation of the parasite immune survival mechanism of antigenic variation. Thus, our work reveals the broad range of activities contributed by RNA–DNA hybrids to *T. brucei* biology, including new functions in host immune evasion as well as activities likely fundamental to eukaryotic genome function.

## Graphical Abstract

**Graphical Abstract:**
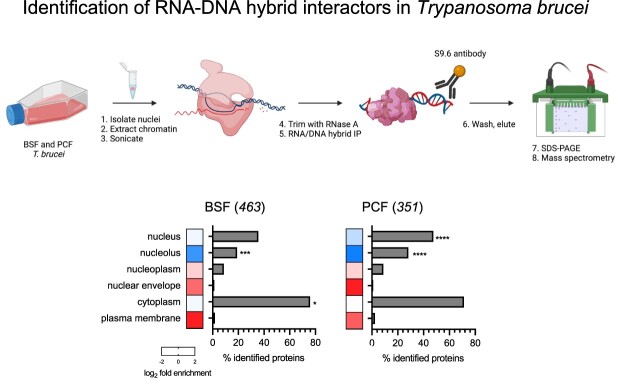

## Introduction

RNA–DNA hybrids are ubiquitous features of genomes in all domains of life. R-loops are a form of RNA–DNA hybrid in which an RNA molecule base-pairs with one strand of double-stranded DNA, causing displacement of a DNA single-strand. Though initially found to form during transcription (1), R-loops are increasingly known to be widespread in genomes and to have wide impacts on genome function, both positive and negative (2–4). Such activities include initiation and arrest of DNA replication (5–7), transcription activation and termination (8), chromatin formation (9,10), and telomere function (11,12). R-loops can result in genome instability (1,13,14), at least in part by generating DNA breaks, which can be harmful (15), but can also be used functionally, such as during class switch recombination in mammalian B cells (16–18). RNA–DNA hybrids and R-loops can also contribute to DNA damage repair, including of DNA double-strand breaks (3,19,20).

A number of studies have recently begun to dissect the various activities provided by RNA–DNA hybrids and R-loops by characterising the proteins with which they interact. The earliest of these studies searched for RNA–DNA hybrid interactors in HeLa cells using immunoprecipitation with the S9.6 monoclonal antibody (21) and mass spectrometry (22), revealing hundreds of potential activities. A similar approach in mouse embryonic stem cells also revealed hundreds of putative RNA–DNA hybrid interactors (23). A distinct approach used two large, synthetic RNA–DNA hybrids and recovered >1000 proteins each from lysates of human B cells (24). Two more recent studies relied on proximity-labelling based on the DNA binding domain of RNase H1, identifying ∼300–400 proteins in immortalised human cells (25,26). Finally, based on all these datasets, Kumer *et al.* (27) searched for common features of the recovered proteins and used machine learning to predict RNA–DNA hybrid interacting proteins across the human proteome. Together, these studies have revealed a wealth of potential RNA–DNA hybrid and R-loop associated activities. However, all these studies are limited to mammals, and no study has asked if similar or distinct activities are found in other eukaryotes. Here, we have adapted the DNA–RNA immunoprecipitation-mass spectrometry (DRIP-MS) approach of Cristini et al (22) to explore the RNA–DNA hybrid interactome of the protozoan parasite, *Trypanosoma brucei*, where mapping of R-loops has predicted both conserved and diverged genomic activities (28).

The genome of *T. brucei* is unusual for a eukaryote in several respects (29,30). All but two of the ∼8000 protein-coding genes in *T. brucei*’s genome ‘core’ (see below) are transcribed by RNA Polymerase (Pol) II from multigene clusters, each of which contains potentially hundreds of genes and is transcribed from a single, still only partly understood transcription start site (31–35). Unlike in bacteria, genes in such operon-like transcription units appear not be functionally related (36). This arrangement means that the genome core contains relatively limited content that is not traversed by RNA Pol-II, and mature mRNAs are generated from pre-mRNA transcripts by extensive, coupled trans-splicing and polyadenylation (37). In addition, each multigene transcription unit has a single transcription termination site, which contains a novel base, termed J, that acts to recruit a number of termination factors (38). This unusual organisation of gene expression appears to also reflect DNA replication organisation, since mapping sites of replication initiation, termed origins, reveals close overlap with transcription start and termination sites (39,40). Furthermore, RNAi against a subunit of the origin recognition complex, which defines origins (41), suggests functional interaction between the replication and transcription machineries (39). All the above aspects of gene expression appear conserved with the wider grouping of kinetoplastids, while other aspects of the *T. brucei* genome may be specific. Survival of African trypanosomes in the mammal relies on a process termed antigenic variation, which involves continuous changes in the identity of a surface expressed ‘coat’, which is composed of a single Variant Surface Glycoprotein (VSG) in a single cell at a given time (42,43). Switching from one VSG coat to another in the mammal relies on both transcriptional changes between approximately 15 telomeric *VSG* transcription sites, termed bloodstream expression sites (BES), and recombination reactions that move silent *VSG* genes into the actively transcribed BES (43,44). Each BES is also a multigene transcription unit but is, remarkably, transcribed by RNA Pol-I from a promoter that shares some homology with those at rRNA gene clusters (45). Recombination relies on a huge archive of 1000s of silent *VSG*s, which are mainly found in arrays that occupy the chromosome subtelomeres (29,46,47). Each chromosome in *T. brucei* thus comprises a highly transcribed core and predominantly untranscribed subtelomeres, with chromosome conformation capture and ATAC-seq analyses indicating that the two genome compartments rarely interact and display differing levels of chromatin-mediated compaction (30).

Previous work has mapped R-loops in *T. brucei*, revealing that their localisation and potential functions span all the above aspects of the genome. DNA–RNA immunoprecipitation and sequencing (DRIP-seq) indicates that R-loops localise to the start and, to a lesser extent, end of the RNA Pol-II multigene clusters, as well as to intra-cluster regions of splicing and polyadenylation (28). The same approach showed that R-loops localise to the single centromere found in each chromosome. Mutation or RNAi has also been used to examine the impact of loss of either *T. brucei* RNase H1 or the A subunit of RNase H2, both of which are RNA–DNA endonucleases that remove RNA from RNA–DNA hybrids (48). Mutation of RNase H1 has no effect on growth, while RNAi of RNase H2A stops growth and results in DNA damage associated with RNA Pol-II transcription initiation; loss of either RNase H results in R-loop and DNA damage accumulation within the *VSG* BESs, as well as loss of VSG expression control and increased *VSG* switching (49–51). Loss or overexpression of RNase H1 or RNase H2A also alters levels of telomeric RNA–DNA hybrids (49,50,52,53), and such telomere-focused changes may also influence VSG expression and switching. To begin to explore this diverse range of RNA–DNA hybrid functions in *T. brucei*, we show here that DRIP-MS recovers a similarly large number of interacting proteins as is observed in mammalian cells. Amongst these putative interactors we can identify functions conserved in mammals, including ribosome- and mRNA-associated factors and helicases, as well as activities that may reflect the unusual *T. brucei* genome, including histone variants and centromere-binding kinetochore proteins. We provide functional analysis of three DRIP-MS factors and show that loss of any of them increases nuclear damage and RNA–DNA hybrid levels, as well as altering VSG expression. One of these factors is a RAD51-related protein previously described to act in *VSG* recombination (54,55), while the two others are putative helicases that have never been functionally examined in *T. brucei*.

## Materials and methods

### Trypanosome culture and genetic editing

*Trypanosoma brucei* MiTat1.2 (Lister 427) bloodstream forms were cultured in HMI-9 medium (Life Technologies) supplemented with 10% foetal calf serum (FCS) at 37ºC, 5% CO_2_, and Lister 427 procyclic forms were cultured in SDM-79 medium with 10% FCS and 0.2% hemin at 27ºC. Bloodstream forms capable of CRSIPR-Cas9 modification were generated by transfection with plasmid pJ1399 (56), containing T7 polymerase and Cas9. Expression of Cas9 was confirmed by RT-PCR (Supplementary Figure S4). For epitope tagging and allele knockout, primers were designed with LeishGEdit (http://www.leishgedit.net/) and donor sequences were PCR amplified from the pPOT plasmid (containing mNeonGreen and blasticidin resistance gene) as previously described (57); PCR products were transfected along with a distinct PCR product to generate guide RNA, which was amplified using self-annealing primers derived also from LeishGedit. Mutant lines were generated by Amaxa transfection with ethanol-purified PCR products and drug-section to generate clonal line, which were confirmed by PCR. Tagged proteins were confirmed by western blot using anti-mNeonGreen antibody (1:1000) and anti-EF1alpha (1:25000) as a loading control. For inducible RNAi, the BSF Lister 427 derivative 2T1 (58) was used. Target sequences and primers were designed with RNAit (59). PCR amplified fragments were cloned into the pRPaiSL vector plasmid (60), which was linearised and transfected to generate RNAi clones, confirmed by RT-PCR.

### RT-qPCR

RNA was extract from 8 × 10^6^ cells using the RNeasy Mini Kit (Qiagen) protocol. Genomic DNA was digested on-column for 15 min at room temperature using the RNase-free DNase I Set (Qiagen). First-strand complementary DNA (cDNA) synthesis cDNA was generated from 500 ng total RNA using SuperScript™ IV First-Strand Synthesis System (Invitrogen), following the manufacturer's protocol using random hexamer primers. Previously used primer sequences were used for VSG RT-qPCR (39). Each primer pair target was run in two biological replicates and three technical replicates for each cell line. A 20 μl reaction contains 1X SYBR. Green PCR Master Mix (Applied Biosystems), 250 nM of forward and reverse primers and 1 μl cDNA. All qPCR experiments were run in 7500 Real-Time PCR system (Applied Biosystems) using the following cycling conditions: 1 cycle at 95ºC for 10 min, followed by 40 cycles of 95ºC for 15 s and 60ºC for 1 min. Fluorescence intensity was measured at the end of each extension step (60ºC for 1 min). For normalization across different samples, actin amplification was used as endogenous control. For calculation of relative mRNA levels, the 2-ΔΔCt method was used (61).

### Fluorescence microscopy

For imaging mNeonGreen fluorescent proteins, ∼8 × 10^6^ parasites were pelleted by centrifugation at 800 rcf for 7 min and resuspended in FCS-free HMI-9 media with 1 μg/ml Hoechst 33342 (Sigma-Aldrich). Cells were pelleted again, resuspended in of 0.05% (v/v) formaldehyde in FCS-free HMI-9 to immobilise parasite flagella and adhered to a Poly-l-Lysine coated slide for immediate imaging. For immunofluorescence analysis of DNA damage, washed parasites were fixed in 4% formaldehyde in vPBS for 10 min and permeabilised with 0.1% IGEPAL CA-630 for 10 min. Cells were washed in vPBS and adhered to a slide, before incubating in PSB + 1% (w/v) glycine 5 min and blocked with 1% bovine serum albumin (BSA) for 1 h. Staining was performed with α-yH2A primary (1:1000) and α-rabbit Alexa Fluor® Plus 488 (1:1000) secondary antibodies diluted in 1% BSA. Slides were washed 1× PBS before mounting with 5 μl DAPI Fluoromount-G® (Southern Biotech) and imaging. For imaging DNA–RNA hybrids, parasites were instead fixed in 70% ice-cold methanol for 1 h, permeabilised with 0.5% v/v Triton X-100 for 10 min and blocked with 1× PBS, 0.01% v/v Tween-20, 0.1% w/v BSA for 1 h at 37ºC. S9.6 (Kerafast) primary (1:1000) and Alexa Fluor Plus 488 Goat anti-Mouse IgG (H + L) (ThermoFisher) secondary (1:3000) antibodies were diluted in blocking solution for staining in suspension while shaking before adhering to slides. VSG immunofluorescence analysis was performed following the protocol of Glover *et al.* (62) Briefly, formaldehyde fixed parasites were adhered to glass slides and blocked with 50% FCS in PBS for 45 min before staining with primary anti-VSG (1:10000) and secondary Alexa Fluor (1:1000) antibodies and mounting with DAPI Fluoromount-G® (Southern Biotech). Imaging was performed with an Axioscope 2 widefield fluorescence microscope (Zeiss) using a 63×/1.40 oil objective, or a Leica DiM8 widefield fluorescence microscope to acquire Z-stacks of 5 μm thickness in 25 sections. The images were later processed on Fiji/ImageJ (63) using the same parameters (http://imagej.net/Rolling_Ball_Background_Subtraction). For fluorescence intensity quantification, all images were obtained using the same exposure times and were later processed on ImageJ using the same parameters. After image processing, a circular 21 × 21 pixel region of interest (ROI) was drawn around each nucleus, and the mean pixel intensity per nucleus was plotted into a vertical scatter plot using Prism 9 (GraphPad).

### DRIP-MS

DRIP-MS approach was adapted from Cristini *et al.* (22). 2.5 × 10^8^ mid-log growth phase parasites were pelleted and washed in 1 ml of 1× PBS, before incubation in 1 ml of lysis buffer (80 mM KCl, 5 mM PIPES pH 8.0, 0.5% Nonidet *P*-40 substitute) on ice for 20 min. The pellet was homogenized using a tight-fitting dounce homogenizer until the nuclei were released, as checked with microscopy. Nuclei were enriched by centrifugation (2400 rcf for 10 min, 4ºC) and resuspended in 200 μl of RSB sonication buffer [1× RSB buffer (10 mM Tris–HCl pH 7.5, 200 mM NaCl, 2.5 mM MgCl_2_), 0.2% sodium deoxycholate, 0.1% SDS, 0.05% sodium lauroyl sarcosinate, 0.5% Triton X-100] before sonicating for 10 min in the Diagenode Bioruptor using cycles of 30 s ON and 90 s OFF. The sonicated samples were then diluted with 600 μl RSB + T buffer (1× RSB buffer, 0.5% Triton X-100) plus 0.8 ng RNase A to degrade single-stranded RNA. For benzonase control IPs, 1 U/μl benzonase was added and incubated for 30 min at 37ºC. 20 μl was reserved as input control samples. Pre-prepared Protein A Dynabeads (incubated with 5 μg of S9.6 antibody (Millipore) in 1× PBS plus 0.5% BSA overnight at 4ºC), were washed in 1× PBS + 0.5% BSA and suspended in the sonicated sample. Samples were incubated on a rotor at 4ºC for 2 h to bind DNA–RNA hybrids and interacting proteins. Beads were then wash in 1 ml cold RSB + T buffer (4 times) and 1 ml cold RSB buffer (twice), before collecting by centrifugation at 1000 rcf for 3 min at 4ºC. Beads were retained using a magnetic rack and supernatant was discarded. Beads were suspended in 15 μl loading buffer (1× NuPAGE LDS loading buffer, 1× PBS, 0.1% β-mercaptoethanol) for 10 min at 70ºC. Beads were pelleted and supernatant, containing eluted proteins collected.

Proteins were resolved using sodium dodecyl sulfate-polyacrylamide gel electrophoresis (SDS-PAGE) and stained using InstantBlue® Coomassie Protein Stain (Abcam). Gel segments only found in S9.6 IP samples and not benzonase controls were excised for Nanoflow HPLC Electrospray Tandem Mass Spectrometry (nLC-ESIMS/MS), performed by Proteomics Facility of Glasgow Polyomics. Briefly, the gel pieces were de-stained by incubation in 500 μl of 50% acetonitrile in 100 mM ammonium bicarbonate for 30 min on a shaker, dehydrated by incubation in acetonitrile for 10 min then dried a vacuum centrifuge. Trypsin was added to rehydrate the gel pieces and digested overnight at 37ºC, before gel pieces were pelleted. The supernatant containing peptides was dried in a vacuum centrifuge and solubilized in 20 μl 5% acetonitrile with 0.5% formic acid.

Trypsinized peptide samples were analyzed using nanoscale liquid chromatography coupled to electrospray ionization tandem mass spectrometry (nLC-ESI–MS/MS). Online detection of peptide ion was by electrospray ionization mass spectrometry using an Orbitrap Elite MS (Thermo Scientific). Peptides were separated on a PepmapTM C18 reversed phase column (3 μm, 100 Å, 75 μm × 50 cm) (Thermo Scientific). Samples were fractionated with mobile phase A consisting of 0.1% (v/v) formic acid in water and mobile phase B consisting of acetonitrile (80% v/v) and water (20% v/v). The peptide separation was performed at a fixed solvent flow rate of 0.3 μl/min, using a gradient of 4–100% mobile phase B over 120 min. The Orbitrap EliteTM MS acquired full-scan spectra in the mass range of m/z 300–2000 Da for a high-resolution precursor scan at a set mass resolving power of 60 000 (at 400 *m*/*z*). Collision-induced dissociation was performed in the linear ion trap with the 20 most abundant precursors using rapid scan mode.

Proteins were identified using the Mascot search engine (v2.6.2, Matrix Science) by interrogating MS data against protein databases of *Trypanosoma brucei* Lister 427. A mass tolerance of 10 ppm was allowed for the precursor and 0.3 Da for MS/MS matching, with the following search parameters: trypsin enzyme specificity, allowing one missed cleavage; cysteine carbamidomethylation was selected as fixed modification, while N-terminal carbamidomethylation, asparagine-glutamine deamidation, tyrosine iodination, and methionine oxidation were selected as variable modifications. Proteins within a significance score of *P* < 0.05 and with at least one unique peptide were considered. Four and two independent biological replicates were generated for BSF and PCF *T. brucei*, respectively. Corresponding emPAI values were compared between IP and benzonase controls; proteins with mean log_2_ fold-change > 0 across all biological replicates constituted the RNA/DNA hybrid interactome for each form. BSF-specific interactome was defined as proteins with > 0 log_2_ fold-change in BSF versus PCF.

### Homology searches, domain analysis and phylogenetics

To look for putative homologs of Tb927.3.2600 and Tb927.3.5440 in Discoba and elsewhere, sequence- and HMM-based similarity searches were done in HMMER (64) and HHpred (65,66). KEGG Orthology (KO) scores, which guided our searches for homologs and phylogenetic analyses, were obtained with KofamKOALA (67). To determine the Pfam domain organisation of every analysed protein, sequences were run through InterProScan (68). Helicase ‘core’ regions were defined according to Fairman-Williams *et al.* (69). The regions of sequence similarity shown in Figure 2, were obtained from HHpred searches using Tb927.3.2600 and Tb927.3.5440 as queries against HMM databases representing the proteomes of *Drosophila melanogaster* and/or *Homo sapiens*; for every protein pair that we considered, we defined their ‘region of similarity’ as that corresponding to their whole alignment as retrieved by HHpred. For the phylogenetic analysis of Tb927.3.2600, we first collected putative Tb927.3.2600 homologs from across Discoba (searches were done in publicly available genomic and transcriptomic datasets), as well as representative orthologs of Tb927.3.2600′s top 4 hits from KofamKOALA, namely DDX60 (KO entry: K20103), MTR4 (K12598), ISE2 (K26077), and HelY (K03727), all of which are members of the Ski2-like family of SF2 helicases (69–71). We then used MAFFT-DASH L-INS-i (72,73) to align each of the 5 groups of protein sequences (Tb927.3.2600 and its putative homologs in Discoba; DDX60; MTR4; ISE2; and HelY) separately; in each case, alignment was followed by manual trimming in Jalview (74), down to the helicase ‘core’ plus short flanking regions (approximately 50–100 aa, depending on sequence conservation within each group). All resulting subsequences were then aligned with MAFFT-DASH L-INS-i, and the obtained alignment trimmed with trimAl (75) using a column gap threshold of 10% (‘gt -0.1′ option). This yielded a ‘final alignment’, which was used as input in an IQ-TREE (76) run to infer a maximum-likelihood phylogenetic tree; the best-fit evolutionary model was selected by ModelFinder (in addition to default ‘standard’ models, several mixture models, e.g. EX_EHO, were included in ModelFinder's search), and both SH-aLRT (77) and UFBoot2 (78) were performed with 1000 replicates each. For the phylogenetic analysis of Tb927.3.5440, a similar protocol was followed. Tb927.3.5440′s top 4 hits from KofamKOALA were ATRX (K10779), ARIP4 (K10876), RAD54 (K10875) and RAD54B (K10877), all of which are members of the Rad54-like grouping within the Snf2 family of SF2 helicases (79). KO’s RAD54 entry confusingly includes a mixture of RAD54 and DRD1 orthologs, which we were able to identify in preliminary trees; because DRD1 is also part of the Rad54-like grouping, we included it in our analysis. Phylogenetic trees were rooted with MAD (80), and visualized and edited in iTOL (81).

## Results and discussion

### Identifying the *T. brucei* RNA–DNA hybrid interactome

RNA–DNA hybrids and associated proteins were enriched through immunoprecipitation (DRIP) using the S9.6 antibody, which recognizes RNA–DNA hybrids at an affinity as low as 0.6 nM (82). Since S9.6 can also recognise double-stranded RNA (83), nuclei were first enriched using homogenization after mild lysis and centrifugation. The released nuclei were then sonicated to fragment the genomic DNA and limit co-precipitation of non-specific proteins during RNA–DNA hybrid recovery. DRIP was then performed on native chromatin, without cross-linking, in the presence of RNase A to deplete nuclear RNA not associated with DNA, and thereby limit DRIP of RNA binding proteins. In addition, half of the nuclear extracts were treated with benzonase prior to DRIP. Benzonase digests all forms of nucleic acids (84), including RNA/DNA hybrids, and so these DRIP samples provided controls for recovery of non-specific proteins by S9.6. DRIPs, plus benzonase controls, were performed for both *T. brucei* procyclic forms (infective to the insect vector; two biological replicates) and bloodstream forms (mammal-infective; four replicates) grown in culture. Figure S1A shows the results of such DRIPs after gel separation, revealing that treatment with benzonase caused loss of many detectable DRIP proteins from both PCF and BSF cells. To characterize the recovered proteins, matched sections of gels for both benzonase-treated and non-treated DRIP samples from all independent replicates were analysed by mass spectrometry (MS).

**Figure 1.**
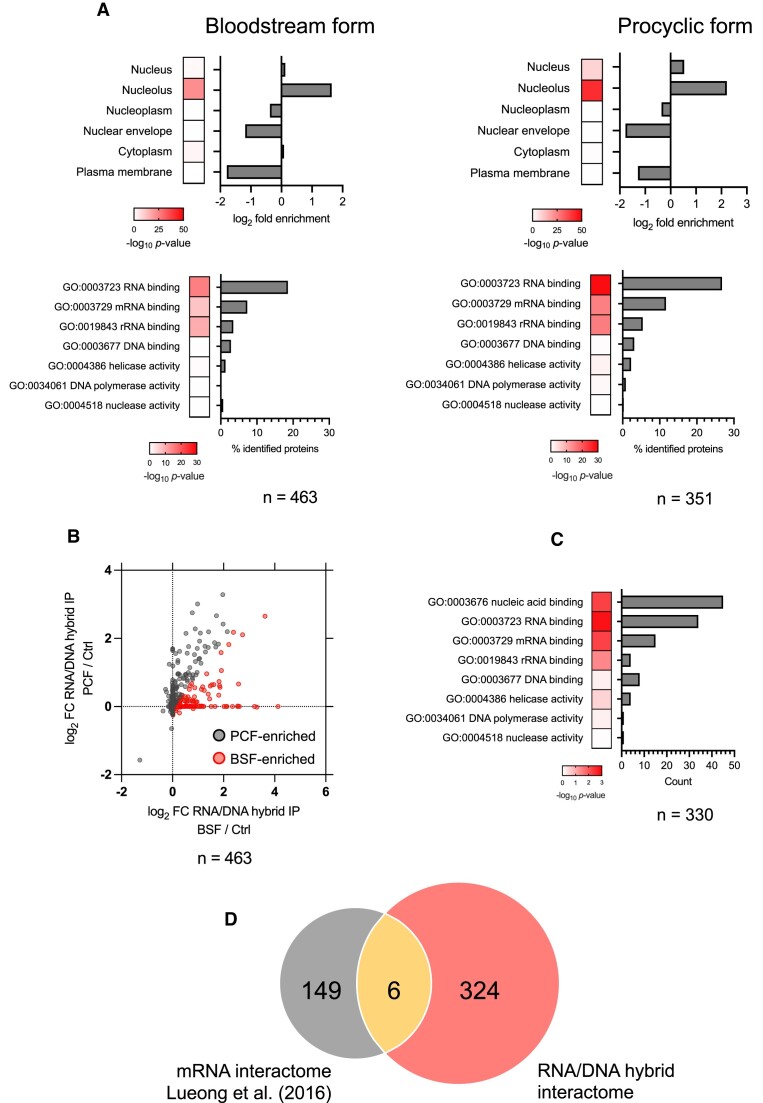
Characterisation of the *T. brucei* RNA–DNA hybrid interactome. (**A**) The top two panels show cellular compartment GO term analysis of proteins recovered by RNA–DNA hybrid immunoprecipitation and mass spectrometry (DRIP-MS) from bloodstream (BSF) and procyclic form cells (PCF), while the lower panels show molecular function GO term analysis of the same datasets. For cellular compartment analysis, fold enrichment relative to the proteome is indicated, while for molecular function analysis the different categories are shown as percentage of total recovered proteins (n); for both, Bonferroni-adjusted p-values are shown as heatmaps. (**B**) Scatter plot of log_2_-transformed mean emPAI values of BSF and PCF DRIP-MS proteins relative to benzonase controls, with those proteins enriched in BSF cells shown in red and those enriched in PCF cells shown in grey. (**C**) Molecular function GO term analysis of BSF-enriched proteins (details as in A). (**D**) Comparison of the BSF RNA/DNA hybrid interactome and mRNA interactome obtained from a study by Lueong *et al.* (163).

To identify putative RNA–DNA hybrid interacting proteins from the label-free DRIP-MS data, emPAI values of all proteins were compared, in each DRIP sample, to protein emPAI values in their cognate benzonase-treated control DRIP samples and log2 enrichment determined. In total, 602 proteins were enriched in at least one DRIP-MS replicate relative to the benzonase controls: 463 and 351 putative RNA–DNA hybrid-interacting proteins in BSF and PCF cells, respectively (Figure 1, Table S1). To ask if the DRIP was selective, fold BSF/PCF enrichment of the 602 DRIP-MS enriched proteins was compared with whole-cell proteomic data from Butter *et al.* (85) (Supplementary Figure S1B). No correlation in BSF/PCF enrichment ratios was seen between these two studies, indicating DRIP enriched for a non-random set of *T. brucei* proteins. Cellular compartment gene ontology (GO) term enrichment analysis, which has been greatly aided by epitope tag-mediated evaluation of the subcellular localisation of most *T. brucei* proteins (86), was next performed on the putative RNA–DNA hybrid interactors. Consistent with nuclear enrichment prior to DRIP, and with the likelihood that most RNA–DNA hybrids will form on the nuclear genome, predicted nuclear and nucleolar proteins showed significant GO term enrichment in the PCF data, and nucleolar proteins were significantly enriched in the BSF data (Figure 1A). In contrast, nuclear envelope and plasma membrane proteins were under-represented. Though cytoplasmic proteins were not enriched (Figure 1A), they represented the majority of proteins recovered from each life cycle stage, perhaps suggesting recovery of proteins that are not RNA–DNA hybrid interactors; for instance, ribosomal proteins were prominent in the DRIP-MS dataset (Supplementary Table S1), possibly due to S9.6 recognition of double-stranded RNA. However, the enrichment of ribosomal proteins may also reflect growing evidence for roles of R-loops in rRNA transcription and processing, leading to ribosome biogenesis (87,88), and is consistent with machine-learning predictions of human RNA–DNA hybrid interacting protein types (27). Moreover, recent work has shown R-loops formed in the nucleus can be found in the cytoplasm of human cells (89), where it is possible that they interact with cytoplasmic proteins. Indeed, below we provide evidence that at least one *T. brucei* RNA–DNA hybrid interactor predominantly localises to the cytoplasm but can provide nuclear activities.

### Conserved eukaryotic RNA–DNA hybrid-associated functions predicted by DRIP-MS

Next, molecular function GO term analysis was used to predict activities of the proteins enriched by DRIP (Figure 1A). Multiple proteins with predicted nucleic acid–binding activities were enriched, mirroring previous interactome data from mammalian cells (22) and consistent with selective S9.6 immunoprecipitation. RNA-binding proteins showed the most significant enrichment in both the BSF and PCF DRIP interactomes. Pronounced enrichment of mRNA-binding proteins is a common feature of our data and that of mammalian HeLa cells (22), and here might reflect the ubiquitous localisation of R-loops with pre-mRNA processing regions in RNA Pol-II polycistrons (28). Enrichment of mRNA binding proteins was non-random (see below) and may suggest the hybrids provide activities linked to mRNA maturation, such as provided by the numerous predicted helicases recovered (Supplementary Table S1). rRNA-binding proteins were also enriched, as also seen by Wu *et al.* in mouse cells (23), providing further evidence of ribosome-associated R-loop functions (Figure. 1A).

A number of classes of enriched factors provide evidence for RNA–DNA hybrids acting during gene expression in *T. brucei*. All four known *T. brucei* histone variants were enriched in the DRIP-MS data (Supplementary Table S1): H2A.Z and H2B.V, which localise at transcription initiation regions, and H4.V and H3.V, which localise to termination regions (33). Thus, the DRIP-MS data reinforces previous *T. brucei* DRIP-seq mapping that showed enrichment of RNA–DNA hybrids at transcription start sites and, to a lesser extent, termination sites, indicating R-loop roles in gene expression organisation (28). In mammalian cells, RNA–DNA hybrids were found to interact with histone H3 (22), and the hybrids are known to recruit histone H3 modifications at promotor regions (90). R-loops have not been mapped in further trypanosomatids but such roles may be conserved, as the histone variants show the same localisations (91,92). How the deposition and functions of trypanosome R-loops and histone variants intersect is unknown, and the potential contribution of the hybrids to transcription remains unclear. However, although the DRIP did not enrich for RNase H1 or RNase H2 (in common with Crisitini *et al.* (22)), loss of RNase H2A causes pronounced DNA damage accumulation at transcription start sites (49). In addition, immunoprecipitation of the histone methyltransferases DOT1A or DOT1B reveals interaction with RNase H2 (32,51), with DOT1A-RNase H2 activity potentially resolving R-loops at transcription termination sites containing RNA Pol-III genes (32). We did not detect either DOT1A or DOT1B in the DRIP-MS data, perhaps suggesting the methyltransferases do not directly interact with RNA–DNA hybrids.

Adding to the above gene expression-associated activities, two of four *T. brucei* (93) Alba proteins were enriched: ALBA3 and ALBA4 (Supplementary Table S1). Alba proteins are found in both archaea and eukaryotes and bind DNA and/or RNA (94). Studies to date have shown *T. brucei* ALBAs to be cytoplasmic and possess RNA binding roles in translation (95–97), and so interaction with RNA–DNA hybrids may be surprising. However, in *Arabidopsis thaliana* ALBA1 binds RNA–DNA hybrids and ALBA2 interacts with the displaced single-stranded DNA, and together acts as an R-loop reader complex (98). Additionally, the four ALBA proteins found in *Plasmodium falciparum* bind both DNA and RNA (99), and so the prediction of RNA–DNA hybrid interaction for *T. brucei* ALBA proteins may indicate previously unknown activities.

DNA-binding proteins showed only weak evidence for enrichment (Figure 1A), unlike the pronounced enrichment seen in mammal DRIP-MS data (22). However, this grouping included some notable factors when specific functions were examined, suggesting roles for *T. brucei* RNA–DNA hybrid interactors in chromosome functions. Previously, we described localisation of R-loops at *T. brucei* centromeres using DRIP-seq (28). Consistent with such localisation, three kinetochore proteins (100) were enriched by DRIP: KKT1, KKT3 and KKT19 (Supplementary Table S1). Though R-loops have also been shown to localise to centromeres in yeast (101), mammals (102) and plants (103,104), it is unclear how they might contribute to, or indeed impede, centromere function. Indeed, most trypanosomatid kinetochore subunits show no evidence of orthology with kinetochores in other eukaryotes (100,105). Intriguingly, *T. brucei* KKT3 is thought to be one of two kinetochore proteins that are positioned most proximal to the centromere (105), where they localise throughout the cell cycle (100). In addition, they contain novel domains for centromere association (106) and recruit KKT1 to assemble the kinetochore (107). DRIP recovery of KKT3 and KKT1 may then suggest that *T. brucei* centromeric R-loops help maintain and guide interaction of the kinetochore to the centromere throughout the cell cycle. Alternatively, R-loops may provide epigenetic definition of the *T. brucei* centromere in the absence of the histone H3 variant, CENP-A (108). SMC (structural maintenance of chromosome) proteins are ATPases found in all domains of life and are core subunits of larger protein complexes needed to organise the genome through conformational change (109). Two of these complexes in eukaryotes are cohesin, which contains Smc1 and Smc3, and condensin, containing Smc2 and Smc4. Here, DRIP enriched for both *T. brucei* SMC2 and SMC4 (Supplementary Table S1). Unlike for cohesin (110,111), no work to date has described condensin functions in any kinetoplastid, and so the prediction of R-loop interaction may provide a route to examine where and how the complex acts in *T. brucei*, where chromosome condensation during mitosis appears to be minimal (112).

Beyond the above protein cohorts, DRIP-MS implicated a number of further, less easy to predict RNA–DNA hybrid-associated activities. For instance, several protein kinases were enriched (Supplementary Table S1), including three NEK family kinases (113), which have diverse roles including in cell cycle control and DNA damage repair (114). Though mitochondrial proteins are likely to be under-represented in our approach, several kinetoplast proteins were enriched (Supplementary Table S1), including two DNA Pols (IC and Beta-PAK) (115,116) and a putative RNA-editing nuclease. Finally, nearly a third of the proteins (180) enriched by DRIP are annotated as hypothetical or hypothetical conserved (Supplementary Table S1), and so no functions can be predicted currently.

### Searching for RNA–DNA hybrid interactor roles in *T. brucei* antigenic variation

Amongst the wealth of potential RNA–DNA hybrid interactors revealed by DRIP-MS, we decided to ask if activities associated with antigenic variation could be identified, since R-loops are involved in the pathway in ways that are not yet clear (117,118). In addition, we reasoned that some R-loop activities that act in antigenic variation could be unique to African trypanosomes and might therefore yield a means to impede this crucial survival mechanism (43).

Despite DRIP-MS not detecting direct RNase H2A or DOT1B interaction with RNA–DNA hybrids, there is clear evidence of functional interaction between these proteins and their involvement in antigenic variation: loss of DOT1B or RNase H2A results in similar changes in R-loop levels, DNA damage levels and Variant Surface Glycoprotein (VSG) expression alterations (49,51). In fact, a connection between R-loops and VSG expression may also extend to the histone variants recovered by DRIP-MS, given similarities in changes to *VSG* transcription control after loss of either RNase H (49,50) or H3.V and/or H4.V (30,119,120). Intriguingly, DRIP-MS data predicted VEX1 as a hybrid interactor, perhaps suggesting even more widespread roles for R-loops in VSG expression (Supplementary Table S1), consistent with the similarity in de-repression of silent *VSG* expression sites after loss or overexpression of VEX1 (62), in RNase H1 mutants (50), and after RNase H2A RNAi (49,51). One explanation for DRIP enrichment of VEX1 may be that the protein localises within or proximal to the telomeres of *VSG* expression sites (121), where RNA–DNA hybrids are present (49,50,52). We did not detect the other components of the VEX complex (121), such as VEX2, however, and so these data may suggest a specific R-loop role of VEX1 (122).

To ask if other RNA–DNA interacting proteins functionally link R-loops and antigenic variation, including by previously unknown mechanisms, we first attempted to compare the abundance of DRIP enriched proteins in BSF and PCF cells, asking which are more prevalent in the former, since VSG is not expressed in the latter (Figure 1B). A total of 330 putative interactors were found to have a greater level of enrichment in BSF cells than PCF relative to benzonase controls. Molecular function GO term analysis of this subset did not reveal any clear difference to all 602 proteins recovered (Figure 1C). Nonetheless, we were now more able to compare the RNA/DNA hybrid interactome with the BSF mRNA proteome generated by Lueong et al (123) (Figure 1D), since the GO term ‘mRNA binding’ was consistently enriched in all DRIP-MS analyses (Figure 1A, C). Overlap between the two proteomic datasets was very limited (Figure 1D), further showing that S9.6 immunoprecipitation recovers a non-random selection of *T. brucei* proteins.

**Figure 2.**
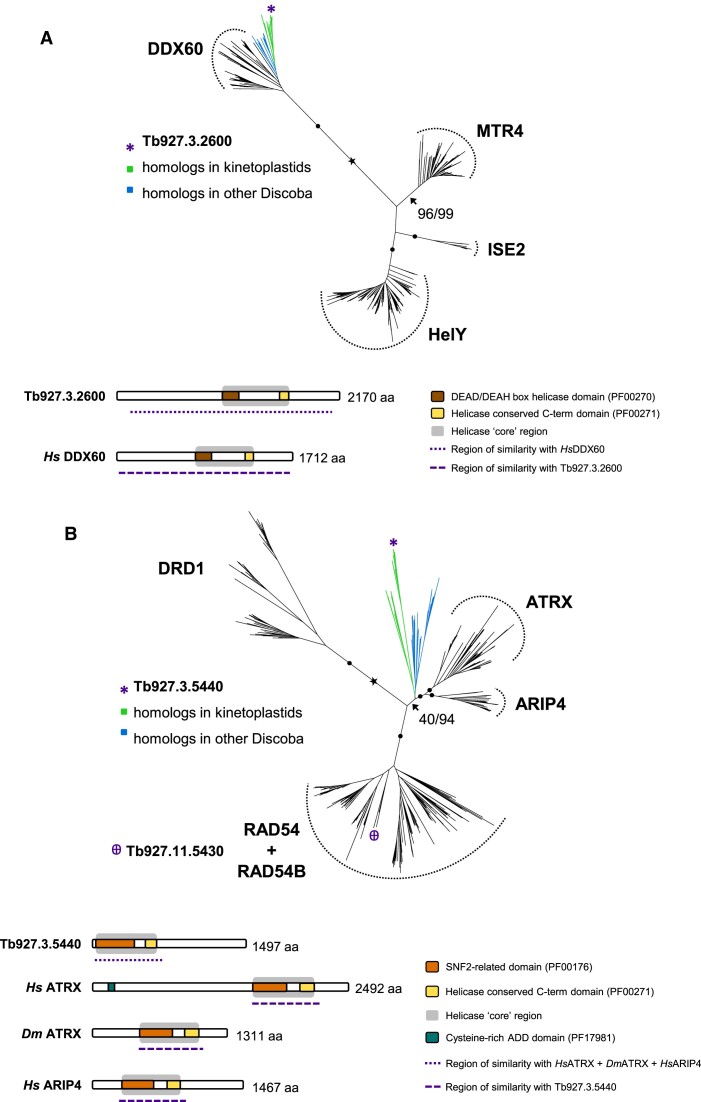
Identification of the putative RNA–DNA hybrid interactors encoded by Tb927.3.2600 and Tb927.3.5440. Phylogenetic and domain analyses are shown for Tb927.3.2600 (**A**) and Tb927.3.5440 (**B**). A maximum-likelihood phylogenetic tree of Tb927.3.2600 homologs found in Discoba and representatives of selected subfamilies within the Ski2-like family of SF2 helicases places Tb927.3.2600 within the DDX60 branch with maximal support; branch support values are SH-aLRT(%)/UFBoot2(%); filled circles represent 100/100 support and the estimated root is indicated with a star. Domain organisation of Tb927.3.2600 and its *Homo sapiens* (*Hs*) homolog DDX60 (UniProt: Q8IY21) reveals that sequence similarity between both proteins extends beyond the helicase core. A maximum-likelihood phylogenetic tree of Tb927.3.5440 homologs found in Discoba and representatives of selected subfamilies within the Snf2 family of SF2 helicases suggests that Tb927.3.5440 does not belong in one of the pictured subfamilies but is likely evolutionary closer to ATRX and ARIP4. Tb927.11.5430 (highlighted) is a *T. brucei* homolog of RAD54/RAD54B. Branch support values and estimated root are as shown for Tb927.3.2600. Domain organisation of Tb927.3.5440 and putative homologs in *H. sapiens* (ATRX, UniProt: P46100; ARIP4, UniProt: Q9Y4B4) and *Drosophila melanogaster* (ATRX, UniProt: Q9GQN5) show that sequence similarity is limited to the helicase core and short flanking regions.

Given that comparing predicted RNA–DNA hybrid interactomes between two life cycle stages did not yield any obvious difference in enrichment patterns, we decided to narrow the search based on two criteria: looking for proteins with annotations of relevant predicted activity; and DRIP-MS indication of recovery only from BSF cells. Amongst the proteins that fulfilled these criteria, four were selected for further analysis. The first two proteins were chosen because they are known to act in *T. brucei* VSG switching: RAD51 (Tb927.11.8190) (124–126) and the RAD51 paralogue, RAD51-3 (Tb927.11.2550) (54,55). RAD51 from yeast and mammals has previously been shown to bind RNA–DNA hybrids (127,128), and we describe the *T. brucei* RAD51-directed connection between R-loops and VSG switching elsewhere (Girasol *et al.*, BioRxiv). RAD51 paralogues are related to RAD51 and provide a range of activities in DNA damage repair and replication (129,130), but no work to date has suggested interaction with RNA–DNA hybrids. The two other proteins, encoded by Tb927.3.2600 and Tb927.3.5440 (Figure 2), were chosen as they provide relevant predicted functions (below) that have not been experimentally examined in *T. brucei* to date.

Tb927.3.2600 encodes one of a number of putative ATP-dependent DExD-box RNA helicases (DDXs) recovered by DRIP (Figure 2A, Table S1). DDXs are one grouping of helicases within a larger superfamily (SF2) (69) and, in other eukaryotes, a number of DDXs have been shown to act on R-loops: DDX21, DDX23, DDX38B and DDX41 each limit DNA damage during transcription (25,131–133); DDX19 is a nucleopore-associated factor that can translocate to the nucleus and act with the kinase ATR during transcription-replication clashes (134,135); DDX1 contributes to immunoglobulin class switch recombination (136); and DDX1, DDX5 and DDX18 act on R-loops associated with DNA damage, including through interaction with DNA repair factors (137–140). Many of these helicases were enriched by DRIP-MS from mouse cells (23). Homology searches and phylogenetic analyses strongly suggest the *T. brucei* Tb927.3.2600-encoded DDX to be a homologue of DDX60 (Figure 2A) and a member of the Ski2 helicase family (69), which has not been implicated in R-loop functions in any eukaryote and has only been functionally characterised in *T. brucei* through its putative interaction with the stress response mRNA binding factor, MKT1 (141,142). The syntenically encoded protein in *T. cruzi* has recently been shown to be part of 43S preinitiation complex of the assembling ribosome (143), an association not described in other eukaryotes, including mammals, where DDX60 is non-essential and has been instead implicated in antiviral activities and cancer (144,145). The novelty of *T. cruzi* DDX60 interaction with the ribosome appears to be reflected in structural features not found in its mammalian orthologues (143).

Tb927.3.5440 has been annotated (tritrypdb.org) as encoding a putative SNF2 DNA repair protein, merely suggesting that it belongs to the large Snf2 family of helicases (69) whose members provide a wide range of activities (146,147), including chromatin remodelling, transcription and DNA repair. Homology searches and phylogenetic analyses suggest the protein encoded by Tb927.3.5440 belongs to a somewhat distinct Discoba grouping that is most closely related to ATRX, which is widely distributed in eukaryotes, and ARIP4, which appears limited to animals (Figure 2B). No function has been ascribed to *T. brucei* ATRX or its relative, RAD54/B (encoded by Tb927.11.5430; Figure 2B), which was not detected in the DRIP-MS data (Supplementary Table S1). However, ATRX in other eukaryotes has been shown to have roles in alternative lengthening of telomeres (148–150), in homologous recombination pathway selection (151,152), and in suppression of R-loops in transcribed telomeres (26,153,154). Many of such roles could be consistent with RNA–DNA hybrid functions at the intersection of transcription and recombination in telomeric BESs during VSG switching in *T. brucei*.

### Loss of RAD51-3, DDX60 or ATRX leads to increased nuclear DNA damage

To begin to test the functions of the three predicted RNA–DNA interactors, we engineered MiTat1.2 BSF *T. brucei* cells to permit genetic modification via CRISPR-Cas9 (56). Using these cells, we generated variant parasites expressing each protein as a translational fusion with mNeonGreen (mNG) and, in addition, attempted to make null mutants by replacing each allelic ORF with antibiotic resistance markers.

**Figure 3.**
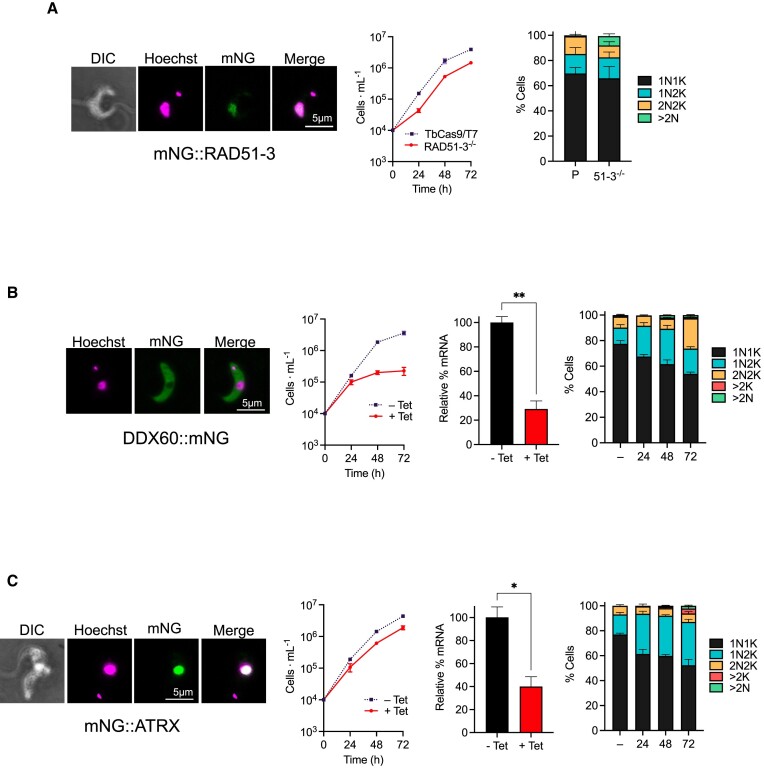
Functional characterisation of *T. brucei* RAD51-3, ATRX and DDX60. For each of RAD51-3 (**A**), DDX60 (**B**) and ATRX (**C**), the following are shown (left to right): representative microscopy images of live fluorescence imaging of a cell expressing the proteins as fusions with mNeonGreen (mNG; scale bar = 5 μm); growth after loss of expression (for RAD51-3 this is a comparison of parental TbCas9T7 cells relative to null mutants (–/–), while for DDX60 and ATRX RNAi induced (+ Tet) and uninduced (– Tet) cells are shown, including relative RNA levels after 24 h of RNAi induction (uninduced RNA level was set at 100%); cell cycle profile of parental and –/– cells (RAD51-3), or before and after RNAi induction for 24, 48 and 72 h (DDX60 and ATRX), as determined through DAPI staining of nucleus (N) and kinetoplast (K). For growth analysis, error bars represent SEM from three independent experiments. For RT-qPCR to determine RNA levels, error bars show SEM from two independent experiments and statistical significance was determined using *t*-test (*P< 0.05). For cell cycle analysis, values are shown as a proportion of cells with specific N–K configurations (1N1K, 1N2K, 2N2K, >2K, others, e.g. >2N) in a cell population (>300 cells); error bars represent SEM from three independent experiments.

Both alleles of all genes were successfully tagged with mNG: RAD51-3 and ATRX at the N-terminus, and DDX60 at the C-terminus (Figures 3 and S2). In each case PCR showed integration of *BSD* and *NEO* constructs, with concomitant loss of the wild type untagged allele, and western blotting using anti-mNG antibody showed expression of fusion proteins of the expected size (Supplementary Figure S2). Tagging of RAD51-3 or DDX60 did not impair parasite growth in culture (Supplementary Figure S2A,B), whereas mNG appeared to at least partially impede ATRX protein function, since the tagged cells exhibited a growth defect compared with parental TbCas9/T7 cells (Supplementary Figure S2C). Live fluorescence microscopy revealed nuclear localization of mNG::RAD51-3 and mNG::ATRX (Figure 3A,C) in all cell cycle stages (Supplementary Figure S2A,C). Fluorescence signal for mNG::RAD51-3 appeared more focal in cells with 1Ne1K and 1N2K compositions of nucleus (N) and kinetoplast (K; elongated, eK) staining (see Figures 3 and S2 for explanation), perhaps indicating recruitment to subnuclear loci during DNA replication (Supplementary Figure S2A). In all cell cycle stages DDX60::mNG signal was cytoplasmic (Figures 3B and S2B), perhaps consistent with a ribosomal function (143). Localisation of each protein in BSF cells essentially matches what is seen in PCF cells (86), suggesting conserved roles in at least these two life cycle stages.

A *RAD51-3* null mutant (*RAD51-3*–/–) was generated in a single round of transfection, with PCR demonstrating replacement of both WT alleles with *BSD* and *NEO*, and RT-PCR showing loss of *RAD51-3* transcript (Supplementary Figure S3A). CRISPR-mediated deletion of *RAD51-3* confirms previous observations that the paralogue is not essential in *T. brucei* (54), which may differ from *Leishmania* (155,156). The absence of RAD51-3 did result in a growth defect, however (Figure 3A), which may be explained by the *RAD51-3*–/– mutants showing an accumulation of cells with more than two nuclei (>2N; 7.4 ± 1.0% in –/–, 0.8 ±0.5% in parental) and a reduction in the proportion of 2N2K cells (9.4 ± 1.7% in –/–, 14 ± 2.9% in parental), suggesting a mitotic defect. Attempts to make null mutants of *ATRX* or *DDX60* failed. For *DDX60*, double antibiotic-resistance transformant clones were recovered, but all retained an intact ORF and displayed improper integration of *BSD* (Supplementary Figure S3B). Attempts to remove even just a single *ATRX* allele failed to yield viable antibiotic-resistant clones. To examine functions, we instead used tetracycline-inducible RNAi (157). For both genes a growth defect emerged from 24 h after RNAi induction, though this was more pronounced for *DDX60* than *ATRX* (Figure 3B, C). Concomitant with the growth defects emerging, DAPI staining revealed perturbation in the DNA content of cells within the populations. For *ATRX*, the most pronounced change was an increase in 1N2K cells (32% in induced, 16% in uninduced at 24 h; Figure 3C), with an associated reduction in 1N1K cells, suggesting a stall in S/G2 phase. After 72 h some cells harbouring more than two kinetoplasts could be seen (4% in induced, 0% in uninduced), indicating kDNA replication and division can occur to at least some extent. The cell cycle defect after *DDX60* RNAi was distinct (Figure 3B), with loss of 1N1K cells associated initially with accumulation of 1N2K cells (24–48 h post-induction) and later by an increase in the proportion of 2N2K cells (72 h). These effects may be explained by death of S/G2-stalled cells from 24–48 h, as there was little increase in cell density at these time points (Figure 3B), or by an S/G2 stall caused by DDX60 loss that is not absolute, with some cells progressing into but not through mitosis. The cell cycle effects described by DAPI staining after RNAi against *DDX60* and ATRX appear consistent with recent analysis of cell cycle perturbations using genome-wide RNAi and characterisation of gene-specific defects by fluorescent cell sorting and next generation sequencing (158).

**Figure 4.**
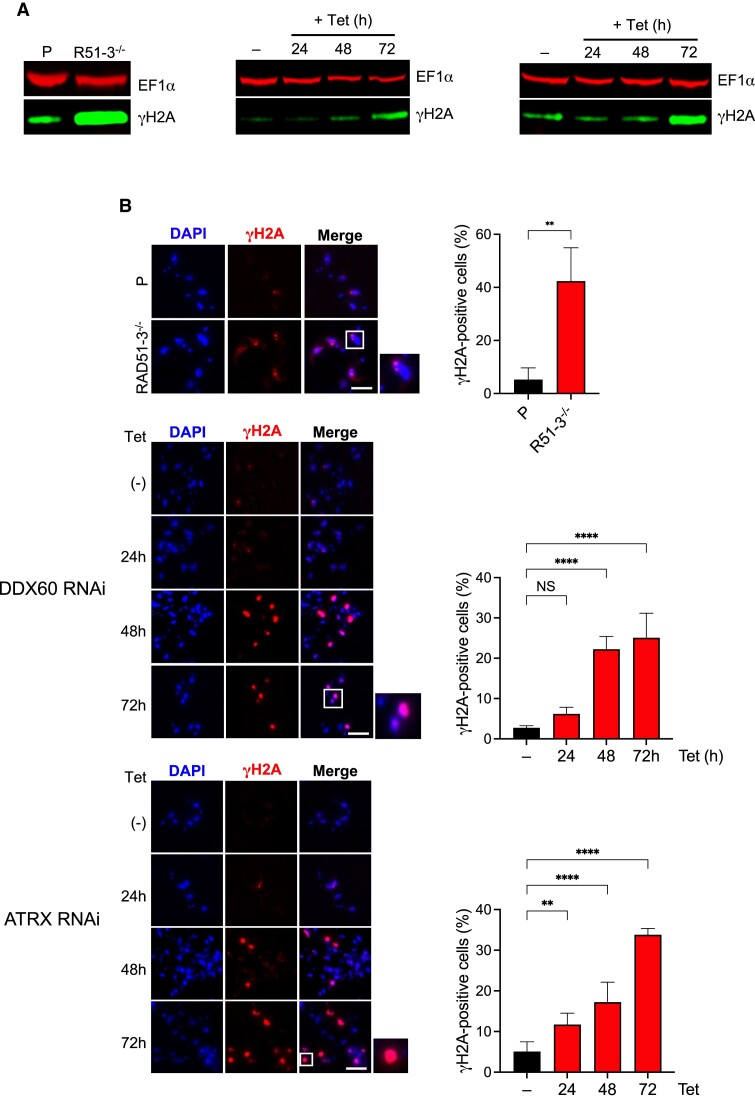
Loss of *T. brucei* RAD51-3, ATRX or DDX60 leads to increased nuclear DNA damage. (**A**) Anti-γH2A western blot comparing *RAD51-3*–/– mutants with parental (P) cells, and before (Tet–) and after *DDX60* or *ATRX* RNAi induction (Tet+) for 24, 48 and 72 h (anti-EF1α was used as a loading control). (**B**) Representative microscopy images of γH2A immunofluorescence in *RAD51-3*–/– mutants and parental (P) cells and before (Tet–) and after *DDX60* or *ATRX* RNAi induction (Tet+) for 24, 48 and 72 h. yH2A localisation is quantified in the adjacent graphs, which show the percent of cells with nuclear anti-γH2A signal. Error bars signify SEM from three independent experiments, counting at least 50 cells; statistical significance was determined for the *RAD51-3*–/– mutants using a *t*-test (**P < 0.01), while statistical significance following RNAi was determined through one-way ANOVA followed by Šídák's multiple comparisons tests (ns, not significant, **P< 0.01, ****P< 0.0001).

To ask if the loss of the three putative RNA–DNA hybrid interactors affects nuclear genome integrity, we tested for levels of Thr130-phoshorylated histone H2A (γH2A), which is a marker for nuclear DNA damage (159). Western blots indicated an increased level of yH2A in *RAD51-3–/–* cells compared with parental, while yH2A levels increased after 72 h of RNAi against *DDX60* or *ATRX* (Figure 4A). To explore these effects further, γH2A was localised and quantified by immunofluorescence (Figure 4B). An increase in the proportion of cells with γH2A-positive nuclei was detected in *RAD51-3*–/– cells (∼42% compared with ∼5% in parental). Moreover, yH2A nuclear signal in the mutants was notably focal (Figure 4B), suggesting discrete DNA damage accumulation and perhaps reflecting the localisation of mNG::RAD51-3 protein (Supplementary Figure S2A). Whether or not these effects are related to replication-associated DNA damage observed after loss of RAD51-3 in *L. major* is unclear (156). Accumulation of nuclear yH2A signal followed the growth defects seen after RNAi of *DDX*60 or *ATRX* (Figure 4B, Figure 3B,C): for the former, no change in the proportion of cells harbouring γH2A signal was seen 24 h after RNAi induction, whereas the signal increased significantly by 48 h and remained essentially the same at 72 h (22% and 25%, respectively); for ATRX, the proportion of cells expressing γH2A increased significantly 24 h post-induction (∼12% in induced, ∼5% in uninduced) and continued to increase from 48–72 h (∼17% to ∼34%, respectively). In both cases, yH2A signal was distinct from that seen in *RAD51-3–/–* cells, in that it appeared throughout the nucleus (Figure 4B). Nonetheless, loss of either of these factors also resulted in nuclear DNA damage, which is perhaps most surprising for DDX60, as localisation of DDX60::mNG suggested it is cytoplasmic (Figures 3 and S2). Whether these data indicate an undetected population of nuclear DDX60, or if the protein can dynamically move between the nucleus and cytoplasm, is unclear.

### Loss of RAD51-3, DDX60 or ATRX alters RNA–DNA hybrid homeostasis

**Figure 5.**
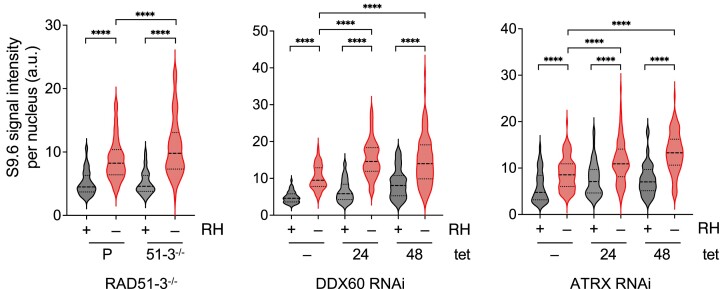
Loss of *T. brucei* RAD51-3, ATRX or DDX60 leads to increased levels of nuclear RNA–DNA hybrids. Violin plots show the intensity of nuclear S9.6 immunofluorescence signal in parental (P) and RAD51-3–/– cells, or before (Tet-) and after *DDX60* or *ATRX* RNAi induction (Tet+) for 24 or 48 h; in all cases the intensity was measured with (+) and without (–) treatment with *E. coli* RNase H1 (RH). Data is shown in each case for >100 cells, the median is shown by a heavily dotted line, and the interquartile range by surrounding lightly dotted lines; statistical significance was determined through one-way ANOVA followed by Šídák's multiple comparisons test (****P < 0.0001).

To ask if loss of the putative interactors affects RNA–DNA hybrid dynamics, we performed immunofluorescence with the S9.6. antibody (Figures 5 and S4). Unlike in mammalian cells (160,161), the majority of anti-S9.6 signal detected in parental or uninduced RNAi *T. brucei* BSF cells was nuclear (Supplementary Figure S4). In addition, and notwithstanding concerns about its effectiveness (161), treatment with *E. coli* RNase H1 significantly reduced S9.6 nuclear fluorescence intensity in the same cells (Figures 5 and S4), indicating much of the signal represents RNA–DNA hybrids, including R-loops.

*RAD51-3–/–* cells displayed significantly increased S9.6 fluorescence compared with the parental cells (Figure 5). In addition, tetracycline induction of the *ATRX* or *DDX60* RNAi cells led to an increase in S9.6 nuclear signal compared to the uninduced (Figure 5). In fact, the temporal changes in S9.6 signal appeared to have parallels with the growth curves (Figure 3B, C) and yH2A immunofluorescence (Figure 4B: for *ATRX*, median fluorescence increased from 24–48 h after RNAi, whereas for *DDX60* median fluorescence increased by 24 h and was unchanged 48 h post-induction (Figure 5). Hence, growth impairment and nuclear DNA damage may follow from increased levels of RNA–DNA hybrids due to the loss of the factors. In addition, the findings reiterate a nuclear function for DDX60. Taken together, these data indicate each of these factors acts in RNA–DNA hybrid homeostasis, consistent with their recovery and identification by DRIP-MS. Whether the altered levels of S9.6 reflect roles for the factors in depositing, resolving or acting upon RNA–DNA hybrids is unclear.

### Loss of RAD51-3, DDX60 or ATRX alters VSG expression

**Figure 6.**
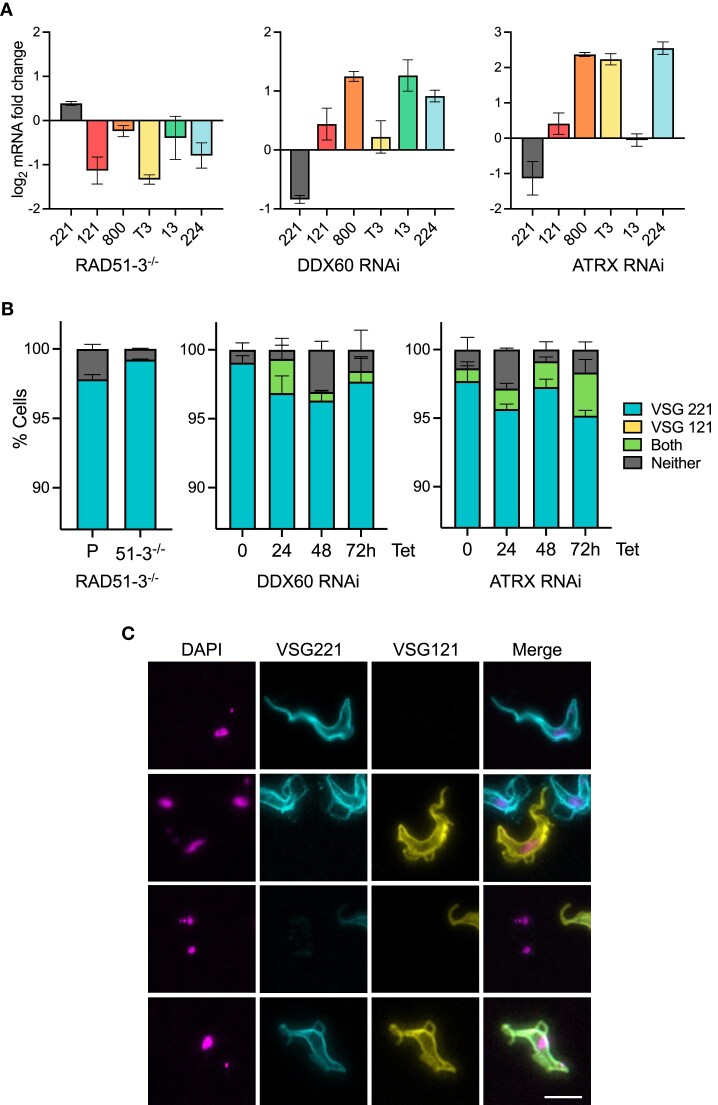
Loss of *T. brucei* RAD51-3, ATRX or DDX60 leads to altered VSG expression. (**A**) Quantification of the relative levels of *VSG* RNAs, comparing parental and *RAD51-3*–/– mutants, or in cells 24 h post-induction of RNAi against *DDX60* or *ATRX* compared with uninduced cells; error bars indicate SEM of two independent experiments. (**B**) Graphical representation of VSG immunofluorescence analysis that shows the proportion of cells staining positive for VSG221 (teal), VSG121 (not detected), both VSGs (green), or neither VSG (grey) in parental (P) and *RAD51-3*–/– cells, or 0, 24, 48 and 72 h after RNAi induction (Tet) against *DDX60* or *ATRX*; error bars represent SEM from three independent experiments, counting >300 cells. (**C**) Sample VSG immunofluorescence images are shown 24 h after *ATRX* RNAi; scale bar = 10 μm.

Given the above evidence linking RAD51-3, DDX60 and ATRX with homeostasis of nuclear RNA–DNA hybrids and with nuclear DNA damage, we next asked if their loss has an impact on VSG switching. MiTat1.2 BSF cells (the strain used for all experiments here) predominantly express VSG221 (also named VSG2) from BES1 [39,40,42]. When wild type MIT1.2 BSF cells are grown in culture, a small proportion (∼1–3%) of cells switch off expression of VSG221 and activate a distinct VSG [39–41,66,67]. To ask if this stochastic switching frequency is altered by loss of the RNA–DNA hybrid interactors, RT-qPCR was first used to assess RNA levels of *VSG221* and four *VSG*s in normally silent BESs (Figure 6A). In the *RAD51-3*–/– parasites RT-qPCR indicated increased levels of *VSG221* transcript relative to parental cells, and an associated reduction in all *VSG* transcripts from the mainly silent BESs. RNAi of *DD60X* or *ATRX* had the opposite effect: in both cases less *VSG221* transcript was expressed in the 24 h induced populations relative to uninduced, and four or five of the silent BES *VSG* transcripts increased in abundance. These data suggest that loss of RAD51-3 reduces switching away from *VSG221* towards any of the silent BES-resident *VSG*s tested, while loss of DDX60 or ATRX increases switching away from *VSG221* and increases activation of the silent BES *VSG*s. Notably, in the latter cases, switching alteration was detected prior to the significant accumulation of DNA damage or pronounced growth defects beyond 24 h of RNAi induction.

To test the effects on *VSG* expression further, we performed immunofluorescence on live cells with antiserum against VSG221 and VSG121, which is expressed from the predominantly silent BES3 (Figure 6B). Consistent with the RT-qPCR, more *RAD51-3*–/– cells that express VSG221 were detected in the population compared to the parental line. In addition, while VSG121 was not detected in parental or mutant populations, a reduced number of *RAD51-3*–/– cells were found that did not express either coat protein. These findings are consistent with previous reports that measured VSG switching using *in vivo* immune selection against VSG221 (54,55), and confirm that RAD51-3 loss reduces the efficiency of *T. brucei* VSG switching. Loss of *RAD51* also leads to decreased VSG switching but, distinct from *RAD51-3* mutants, levels of RNA–DNA hybrids are reduced (Girasol et al, BioRxiv). Hence, more work is needed to understand how the patterns of global and local R-loops are influenced by HR factors, including in the BESs. VSG221 and VSG121 immunofluorescence provided a fuller explanation of the RT-qPCR analysis after *DDX60* or *ATRX* RNAi (Figure 6B). Here, the analysis was conducted over 72 h after RNAi induction and, in both cases, cells were detected that expressed both VSG221 and VSG121 as early as 24 h (an example is shown in Figure 6C). Before induction of *DDX60* RNAi no such cells were detected, whereas a small number were seen prior to induction of *ATRX* RNAi and their numbers increased after induction. Taken together, these findings indicate that loss of either factor impairs the gene expression controls that normally operate to ensure only a single *VSG* BES is transcribed, or their RNAi delays the process of transition from expressing VSG221 to VSG121 (and potentially to any new VSG), as seen after loss of DOT1B (162). These same effects - loss of VSG expression control and altered or increased VSG switching - are also seen after loss of RNase H1 or RNase H2A (49,50), where R-loop levels increase in the *VSG* BESs, indicating a potential link in terms of R-loop homeostasis.

## Conclusions

RNA–DNA hybrids are ubiquitous epigenetic features of all DNA genomes, where their list of functions continues to expand. Understanding this range of functions can be aided by describing the proteins that interact with RNA–DNA hybrids, though such studies have to date only been conducted in mammals. Here, we describe a large cohort of putative RNA–DNA hybrid interactors in the protozoan parasite, *Trypanosoma brucei*, where R-loops have been mapped genome-wide and implicated in both conserved and lineage-specific activities (28). Consistent with predictions of conserved activities, we find overlap between our data and mammalian studies (22,27), including ribosome-associated proteins and predicted RNA helicases. In fact, our data provide a potentially novel link between these activities, in that we describe a predominantly cytoplasmic DDX (DDX60) that has been found to associate with the ribosome in *T. brucei*’s relative, *T. cruzi* (143) and influences RNA–DNA hybrid levels in the parasite and, moreover, appears to have moonlighting activities in nuclear DNA repair. Our data also provides a number of predicted RNA–DNA hybrid interactors with activities that may reflect particular features of trypanosome gene expression and chromosome biology, including histone variants (33) and kinetochore components (100), though it should be noted that tests are needed to determine that these factors do indeed bind RNA–DNA hybrids.

A crucial activity used by *T. brucei* to survive in its mammalian host is antigenic variation, which is driven by transcriptional controls and recombination of *VSG* genes (43). Amongst the proteins recovered by DRIP-MS, we have found four that act in VSG switching. Two are key determinants of DNA repair by homologous recombination: RAD51 (Girasol *et al.*, BioRXiv) and RAD51-3. Though both proteins have previously been shown to act in VSG switching by recombination (44), their interaction with RNA–DNA hybrids provides new mechanistic understanding and builds upon recent work that has implicated R-loops in the reaction (49,50,53,118). Two other factors, DDX60 and ATRX, have never before been implicated in VSG switching. How these factors act in antigenic variation is not yet clear, but the observation that their loss, like that of RAD51-3, leads to increased RNA–DNA hybrids levels, while they have distinct effects on VSG switching to the RAD51 paralogue, illustrates that emerging links between R-loops and VSG transcription or recombination deserve further analysis. For instance, the increased levels of RNA–DNA hybrids seen in *RAD51-3* mutants perhaps indicates DNA repair functions that operate more widely than localised activities dedicated to *VSG* recombination.

## Supplementary Material

gkad836_Supplemental_Files

## Data Availability

DRIP-mass spectrometry proteomics data have been deposited to the ProteomeXchange Consortium via the PRIDE partner repository, with the dataset identifier PXD042146.
