# Supplementary material for: Immunoprecipitation of RNA–DNA hybrid interacting proteins in Trypanosoma brucei reveals conserved and novel activities, including in the control of surface antigen expression needed for immune evasion by antigenic variation
Source: Nucleic Acids Res. 2023 Oct 16;51(20):11123–41. doi: 10.1093/nar/gkad836 (PMC10639054; doi:10.1093/nar/gkad836)
Supplement: gkad836_Supplemental_Files [file gkad836_supplemental_files.zip › Supplementary material.pdf]

Supplementary figure legends

Figure S1. DRIP-MS enriches for a subset of proteins from the total cell proteomes of both BSF and PCF *T. brucei.* (A) Representative Coomassie-stained gel of eluted proteins from DRIP with or without benzonase treatment. BSA indicates Bovine Serum Albumin used to block protein A dynabeads; IgH and IgL indicate heavy chain and light chain of the S9.6 antibody, respectively. Red boxes indicate regions excised for mass spectrometry. (B) Comparison of log2 fold enrichments of proteins in the combined RNA-DNA hybrid interactomes (this study) and the total cell proteome obtained from the dataset in a study by Butter et al (2013).

Figure S2. Localisation of *T. brucei* RAD51-3, ATRX and DDX60. For each of RAD51-3 (A), DDX60 (B) and ATRX (C), the following are shown: PCR showing transformants with both alleles tagged with mNeonGreen (mNG; P, parental TbCas9/T7; Tag, mNG transformant; WT, untagged allele); annealing sites of primers used for PCR; western blot analysis of whole-cell protein extracts probed with anti-mNeonGreen antibody, anti-EF1α antibody was used as a loading control (expected sizes of the mNG-tagged proteins are shown); growth analysis of the mNG transformant compared to the parental TbCas9/T7 cell line (error bars represent SEM from three independent experiments); and representative microscopy images of live fluorescence imaging of mNG transformant cells at different cell cycle stages (scale bar = 5 μm).

Figure S3. Attempts to make null mutants of *RAD51-3* and *DDX60* by CRISPR-Cas9. (A) PCR of a *RAD51-3-/-* transformant and parental (P) TbCas9/T7 cells, demonstrating the expected integration of *NEO* and *BSD* knockout constructs and the corresponding loss of the *RAD51-3* ORF, as well as RT-PCR results demonstrating the loss of *RAD51-3* RNA (*Cas9* was used as a control). (B) PCR to analyse integration of NEO and BSD knockout constructs in a number of *DDX60* transformant relative to parental TbCas9T7 cells; sizes of PCR products for allele replacement integrations are shown, as well as for the unaltered allele (WT), and approximate annealing sites of primers used for PCR are indicated.

Figure S4. Loss of *T. brucei* RAD51-3, ATRX or DDX60 leads to increased levels of nuclear RNA-DNA hybrids. Representative images of S9.6 immunofluorescence, comparing *RAD51-3-/-* and parental TbCas9T7 cells, and cells grown with (Tet-) or without (Tet+) RNAi induction against *DDX60* or *ATRX*; in all cases, images are shown with or without *E. coli* RNase H1 (RH) treatment. Inset images depict zoomed-in regions showing nuclear distribution of R-loops. Scale bar before zoom = 10 μm.

Table S1. Analysis of proteins recovered by DNA-RNA immunoprecipitation and identified by mass spectrometry, showing four bloodstream (BSF; including an RNase H1 mutant, RH1 KO, and a cell

expressing myc-tagged RNase H1, RH1 tag) and two procyclic form (PCF) experiments relative to two and one, respectively, benzonase treated controls (ctrl).

A

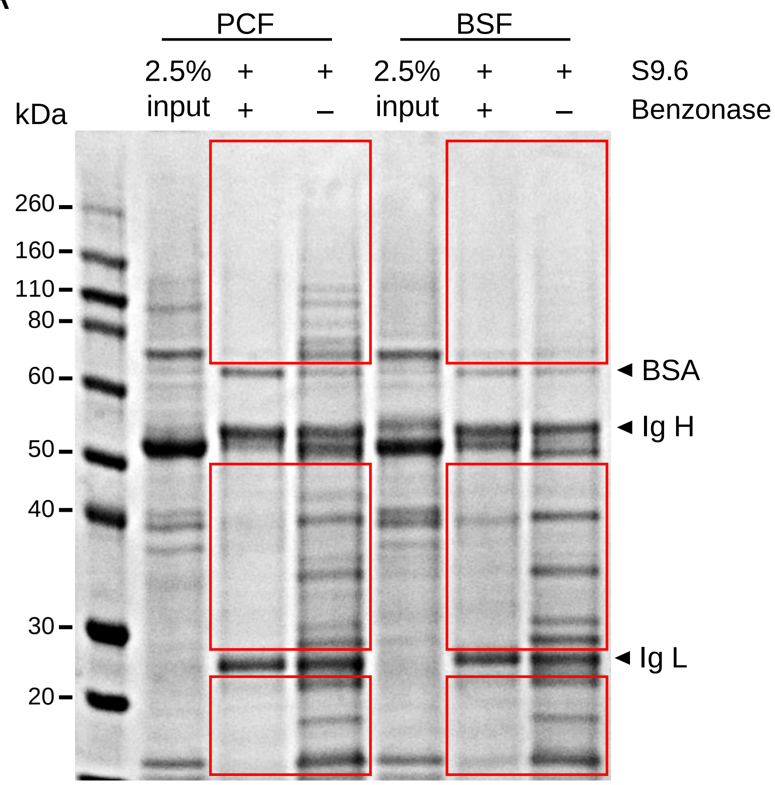
PCF
BSF
2.5% input
+
+
+
–
S9.6
Benzonase
kDa
260
160
110
80
60
50
40
30
20
BSA
Ig H
Ig L

B

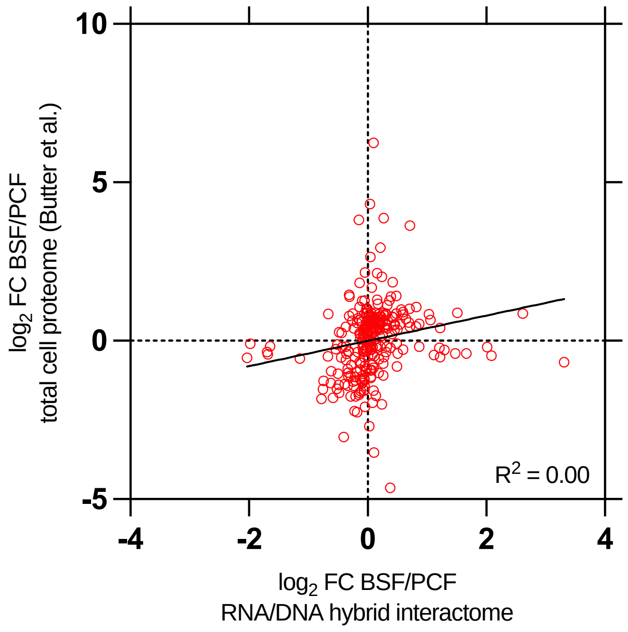
10
5
0
-5
-4
-2
0
2
4
log2 FC BSF/PCF
total cell proteome (Butter et al.)
log2 FC BSF/PCF
RNA/DNA hybrid interactome
R2 = 0.00

Fig. S1

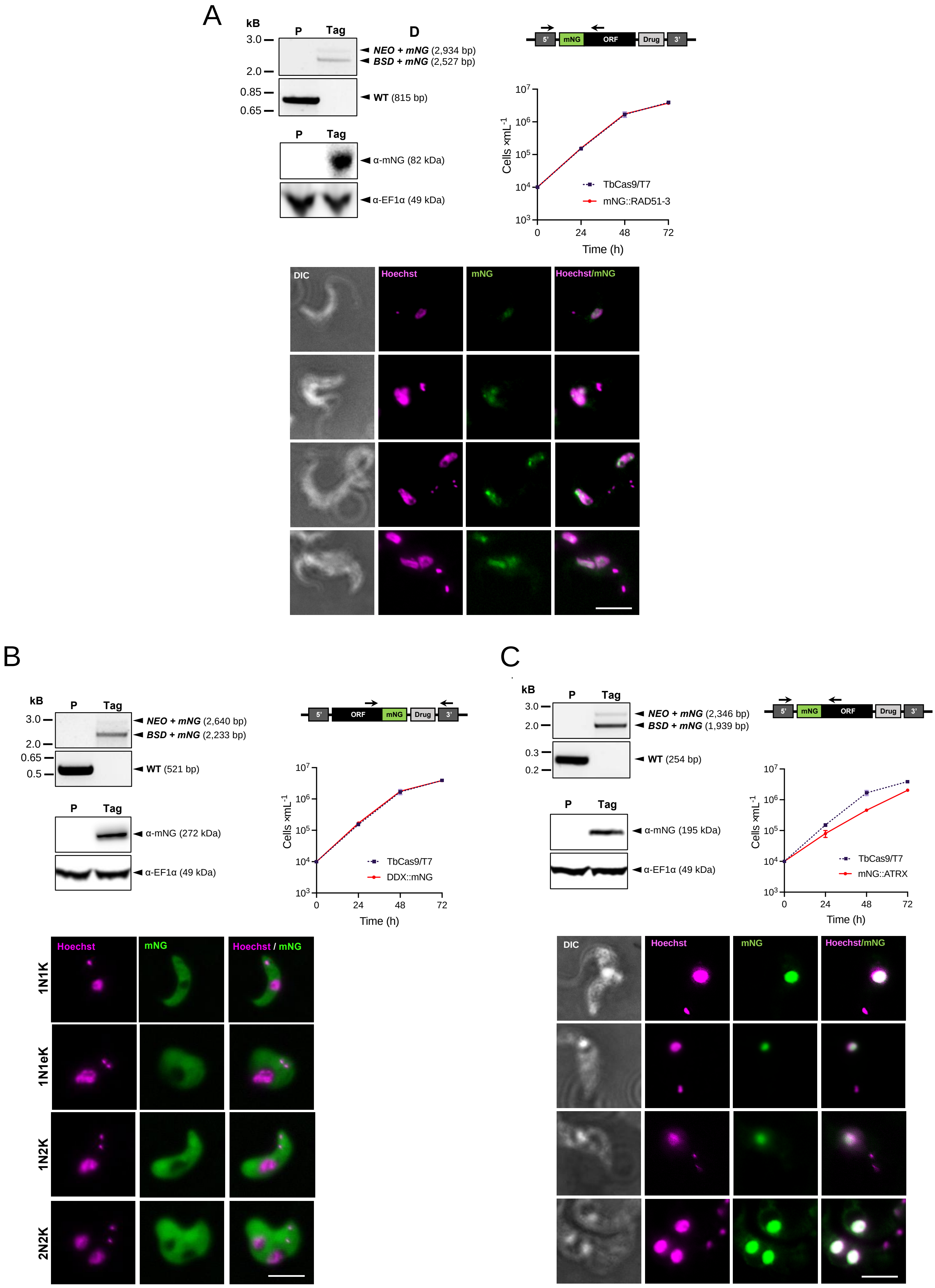
A
kB
P
Tag
D
3.0
2.0
0.85
0.65
NEO + mNG (2,934 bp)
BSD + mNG (2,527 bp)
WT (815 bp)
α-mNG (82 kDa)
α-EF1α (49 kDa)
5'
mNG
ORF
Drug
3'
Cells ×mL-1
Time (h)
TbCas9/T7
mNG::RAD51-3
DIC
Hoechst
mNG
Hoechst/mNG
B
NEO + mNG (2,640 bp)
BSD + mNG (2,233 bp)
WT (521 bp)
α-mNG (272 kDa)
DDX::mNG
1N1K
1N1eK
1N2K
2N2K
Hoechst / mNG
C
NEO + mNG (2,346 bp)
BSD + mNG (1,939 bp)
WT (254 bp)
α-mNG (195 kDa)
mNG::ATRX

Fig. S2

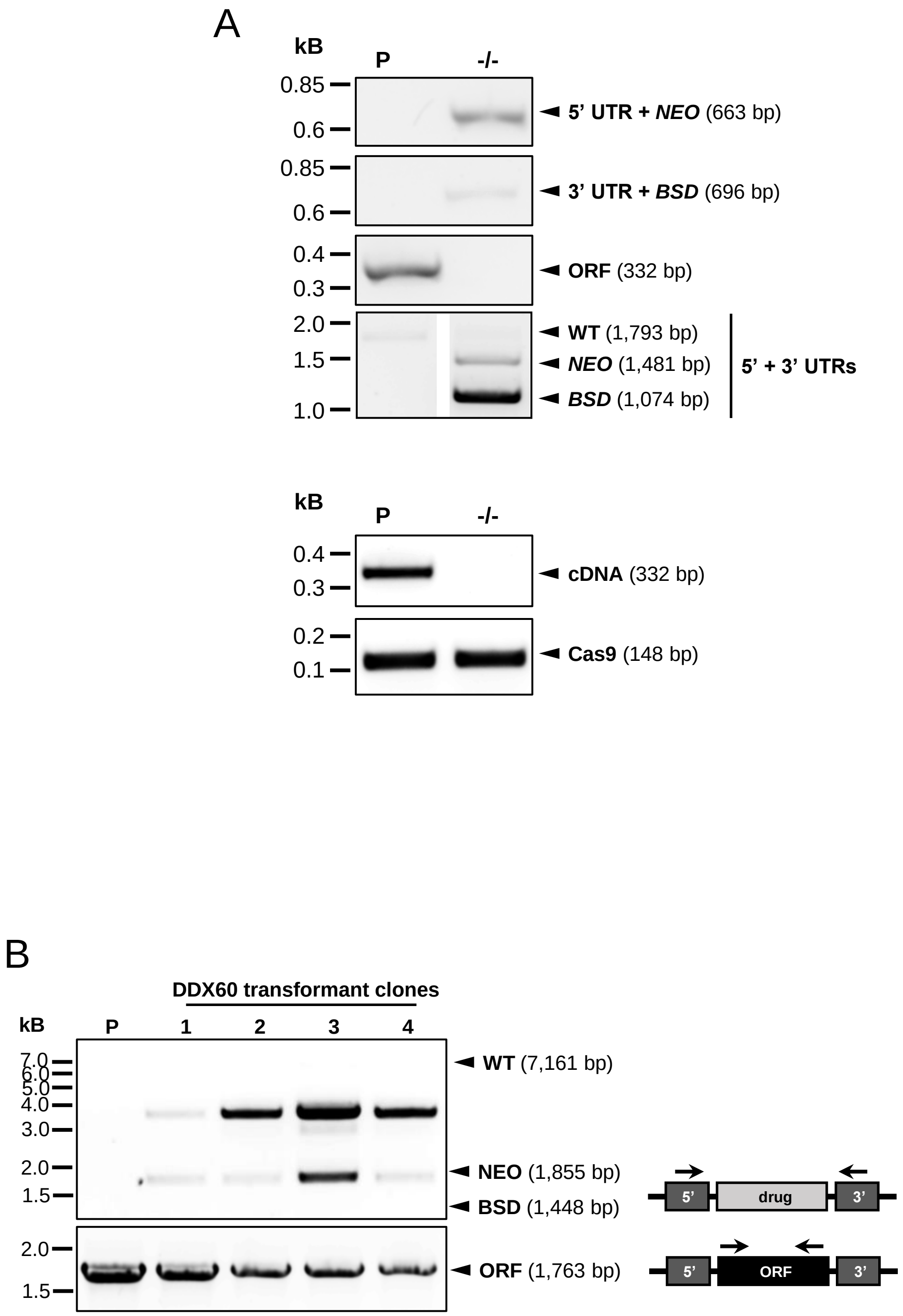
A
kB
P
-/-
0.85
0.6
5’ UTR + NEO (663 bp)
0.85
0.6
3’ UTR + BSD (696 bp)
0.4
0.3
ORF (332 bp)
2.0
1.5
1.0
WT (1,793 bp)
NEO (1,481 bp)
BSD (1,074 bp)
5’ + 3’ UTRs
kB
P
-/-
0.4
0.3
cDNA (332 bp)
0.2
0.1
Cas9 (148 bp)
B
DDX60 transformant clones
kB
P
1
2
3
4
7.0
6.0
5.0
4.0
3.0
2.0
1.5
WT (7,161 bp)
NEO (1,855 bp)
BSD (1,448 bp)
2.0
1.5
ORF (1,763 bp)
5’
drug
3’
5’
ORF
3’

Fig. S3

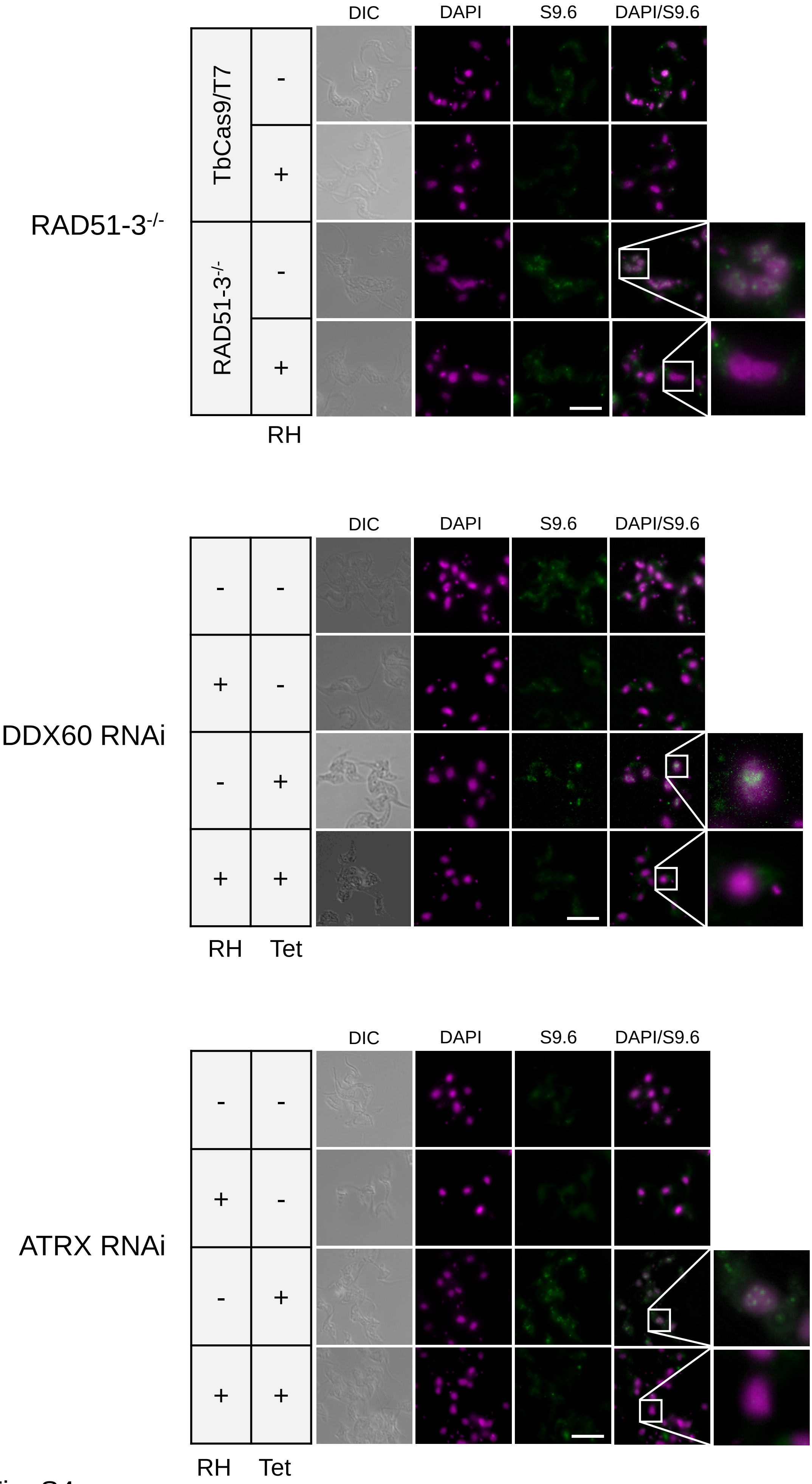

DIC
DAPI
S9.6
DAPI/S9.6
RAD51-3-/-
TbCas9/T7
RAD51-3-/-
-
+
-
+
RH
DIC
DAPI
S9.6
DAPI/S9.6
DDX60 RNAi
-
-
+
-
-
+
+
+
RH
Tet
DIC
DAPI
S9.6
DAPI/S9.6
ATRX RNAi
-
-
+
-
-
+
+
+
RH
Tet

Fig. S4
